# Improving Nursing Students’ Attitude Toward Aging Through Academic and Community Partnerships

**DOI:** 10.1093/geroni/igaf122.2948

**Published:** 2025-12-31

**Authors:** Angela Braswell

**Affiliations:** Wentworth Douglass Hospital, Portsmouth, New Hampshire, United States

## Abstract

“Maybe the time to learn old age’s lessons is in advance. The lessons are out there. Six million teachers are in it now” (Leland, 2018, p. 22). For this 15-week undergraduate nursing course aimed to promote healthy aging and explore nursing issues related to older adults, 94 students engaged in an intergenerational learning opportunity with volunteer “consultants” from a local Continuing Care Retirement Community (CCRC). Using the 4-Ms Framework for Age-Friendly Health Systems and John Leland’s “Happiness is a Choice You Make: Lessons From a Year Among the Oldest Old” for inspiration, students and their consultants met on average five times to complete course assignments, culminating in a video presentation that shared the consultant’s story, highlighted students’ key takeaways, and explained how the experience will inform their future nursing practice. What lessons did they learn? Narrative data and word cloud analysis showed improved student attitudes towards older adults, enhanced empathy, and a deeper appreciation for essential gerontological nursing competencies. The project promoted the growth of transferable essential skills in communication, assessment, and ethical and clinical decision-making. Feedback from consultants highlighted mutual learning, increased connection with younger generation, and satisfaction having contributed to the education of future nurses. Partnerships with local CCRCs offer valuable real-world experiences and a competency-based approach to improve student attitudes toward aging, thereby enhancing health outcomes for older adults. Additionally, these academic and community partnerships can further foster interdisciplinary collaborations, enriching student educational experiences and broadening the program’s impact on community health and well-being.

